# Lup-20(29)-en-28-ol-3-one (betulone)

**DOI:** 10.1107/S1600536813011008

**Published:** 2013-04-27

**Authors:** Stanisław Boryczka, Ewa Michalik, Joachim Kusz, Maria Nowak, Elwira Chrobak

**Affiliations:** a Department of Organic Chemistry, Medical University of Silesia, Sosnowiec 41-200, Poland; b Department of Organic Chemistry, Wrocław Medical University, Wrocław 50-556, Poland; c Department of Physics of Crystals, Institute of Physics, University of Silesia, Katowice 40-007, Poland; d Department of Solid State Physics, Institute of Physics, University of Silesia, Katowice 40-007, Poland

## Abstract

The asymmetric unit of the title compound, C_30_H_48_O_2_, contains two independent mol­ecules, the main difference between them being that the isopropenyl group is rotated by approximately 180°. In each mol­ecule, the fused six-membered rings have chair–chair–chair–chair conformations and the cyclo­pentane ring adopts an envelope conformation with the C atom bearing the hy­droxy­methyl group as the flap. All ring junctions are *trans*-fused. With the exception of one of the methyl groups adjacent to the C=O group, all the methyl groups are in axial positions. The isopropenyl group is equatorial and the hy­droxy­methyl group is in an axial orientation. In the crystal, weak C—H⋯O inter­actions link the mol­ecules into chains along [010]. Weak intra­molecular C—H⋯O hydrogen bonds are also observed but the hy­droxy groups are not involved in hydrogen bonds.

## Related literature

For the synthesis of betulone, see: Hase *et al.* (1981 ▶). For the isolation of betulone from plants, see: Cole *et al.* (1991 ▶); Reyes *et al.* (2006 ▶); Diouf *et al.* (2009 ▶); Liu *et al.* (2010 ▶); Kim *et al.* (2002 ▶); Garcez *et al.* (2003 ▶); Fuchino *et al.* (1996 ▶). For the biological activity of betulone, see: Alakurtti *et al.* (2010 ▶); Hata *et al.* (2002 ▶); Reyes *et al.* (2006 ▶). For related structures, see: Mohamed *et al.* (2006 ▶); Ding *et al.* (2009 ▶); Drebushchak *et al.* (2010 ▶); Boryczka *et al.* (2011 ▶, 2012*a* ▶,*b* ▶).
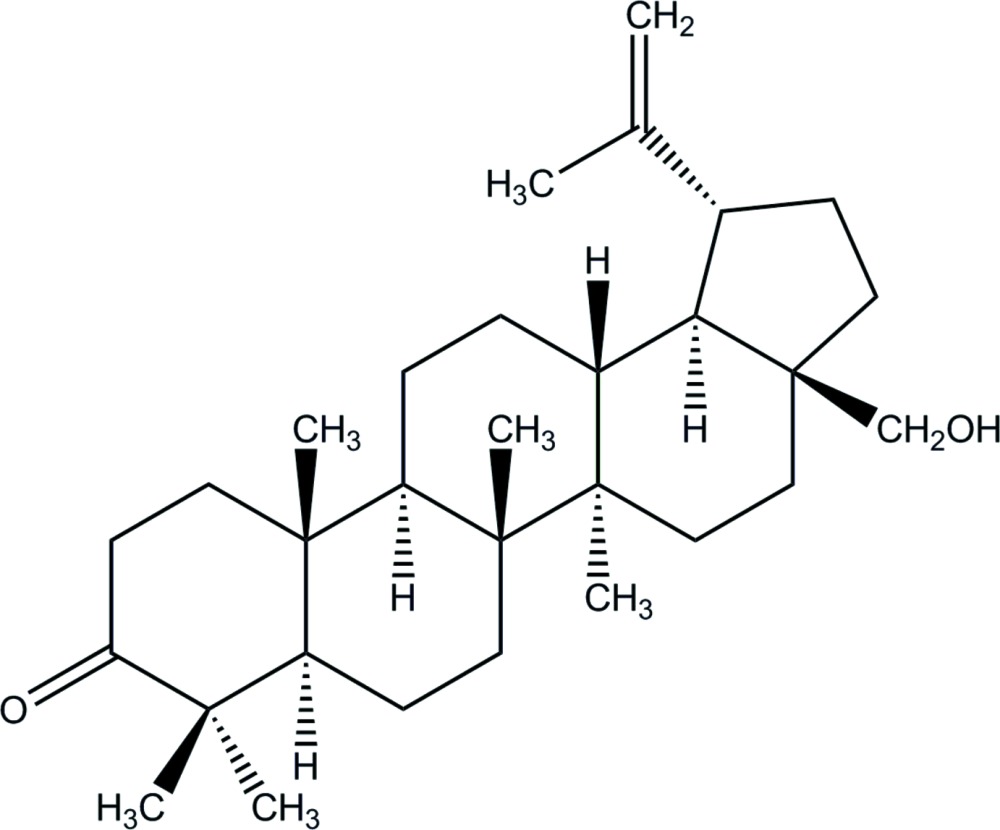


## Experimental

### 

#### Crystal data

C_30_H_48_O_2_
*M* *_r_* = 440.71
Orthorhombic, 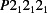

*a* = 9.4447 (3) Å
*b* = 19.1818 (6) Å
*c* = 28.1141 (7) Å
*V* = 5093.3 (3) Å^3^
*Z* = 8
Mo *K*α radiation
μ = 0.07 mm^−1^
*T* = 100 K
0.60 × 0.56 × 0.20 mm

#### Data collection

Oxford Diffraction diffractometer with a Sapphire3 detector
Absorption correction: multi-scan (*CrysAlis RED*; Oxford Diffraction, 2008 ▶) *T*_min_ = 0.960, *T*_max_ = 0.986
61473 measured reflections
5036 independent reflections
4401 reflections with *I* > 2σ(*I*)
*R*_int_ = 0.055

#### Refinement

*R*[*F* ^2^ > 2σ(*F* ^2^)] = 0.051
*wR*(*F* ^2^) = 0.141
*S* = 1.03
5036 reflections
609 parameters
H atoms treated by a mixture of independent and constrained refinement
Δρ_max_ = 0.55 e Å^−3^
Δρ_min_ = −0.50 e Å^−3^

### 

Data collection: *CrysAlis CCD* (Oxford Diffraction, 2008 ▶); cell refinement: *CrysAlis RED* (Oxford Diffraction, 2008 ▶); data reduction: *CrysAlis RED*; program(s) used to solve structure: *SHELXS97* (Sheldrick, 2008 ▶); program(s) used to refine structure: *SHELXL97* (Sheldrick, 2008 ▶); molecular graphics: *Mercury* (Macrae *et al.*, 2006 ▶); software used to prepare material for publication: *publCIF* (Westrip, 2010 ▶).

## Supplementary Material

Crystal structure: contains datablock(s) I, global. DOI: 10.1107/S1600536813011008/lh5603sup1.cif

Structure factors: contains datablock(s) I. DOI: 10.1107/S1600536813011008/lh5603Isup2.hkl

Additional supplementary materials:  crystallographic information; 3D view; checkCIF report

## Figures and Tables

**Table 1:** Hydrogen-bond geometry (Å, °)

*D*—H⋯*A*	*D*—H	H⋯*A*	*D*⋯*A*	*D*—H⋯*A*
C13*A*—H13*A*⋯O2*A*	1.04 (4)	2.52 (3)	3.186 (4)	122 (2)
C13*B*—H13*B*⋯O2*B*	1.02 (3)	2.47 (3)	3.165 (4)	125 (2)
C19*A*—H19*A*⋯O2*A*	0.95 (4)	2.45 (4)	3.006 (5)	118 (3)
C22*B*—H22*C*⋯O1*B* ^i^	1.05 (4)	2.56 (4)	3.567 (4)	160 (3)

Symmetry code: (i) 
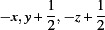
.

**Fig. 1.**
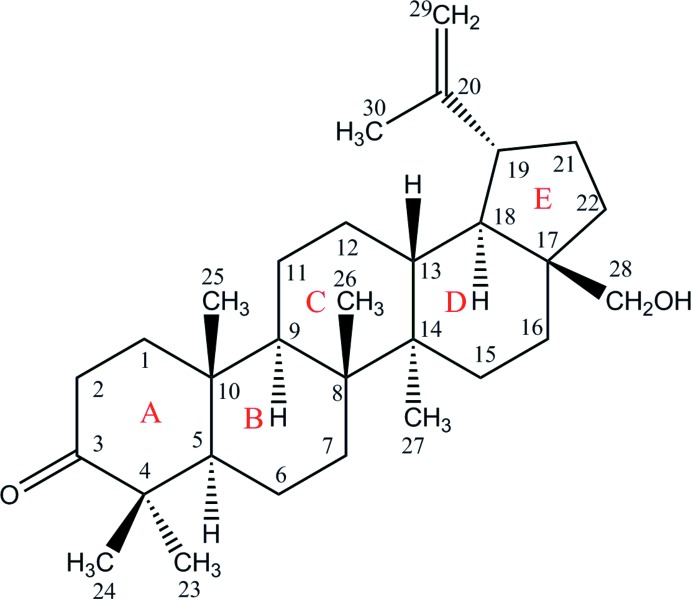
Atom and ring numbering scheme for the title compound.

**Fig. 2.**
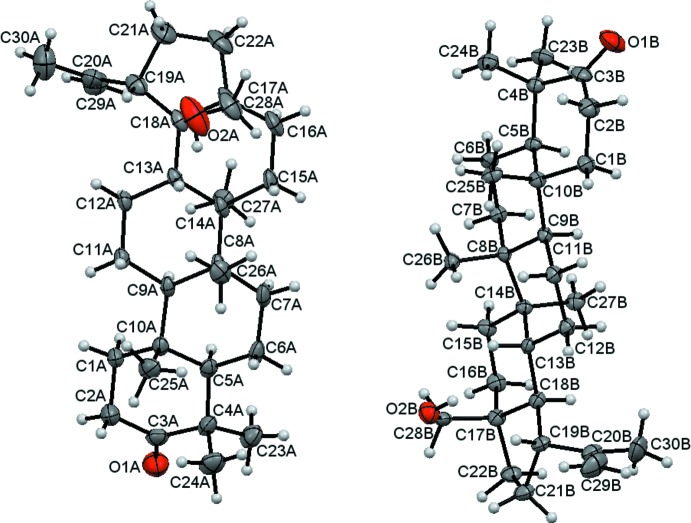
The asymmetric unit of the title compound. Displacement ellipsoids are drawn at the 50% level.

**Fig. 3.**
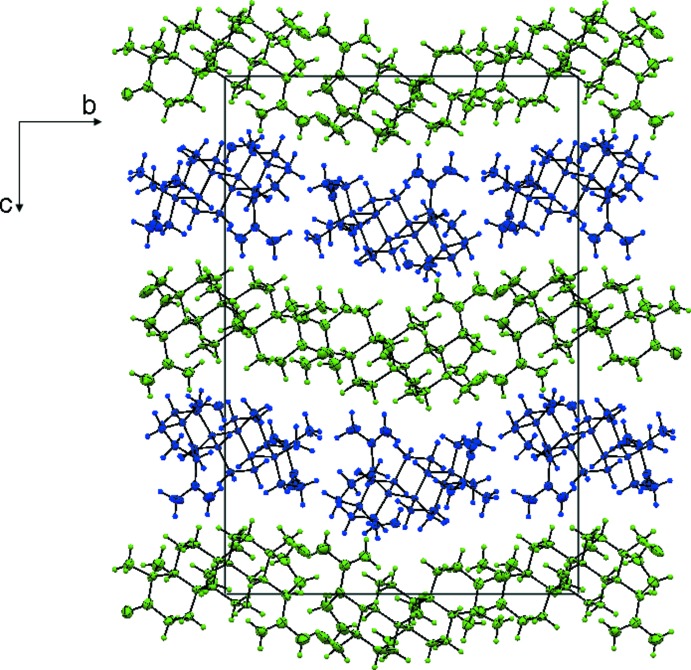
View of the unit cell along the crystallographic a axis.

**Fig. 4.**
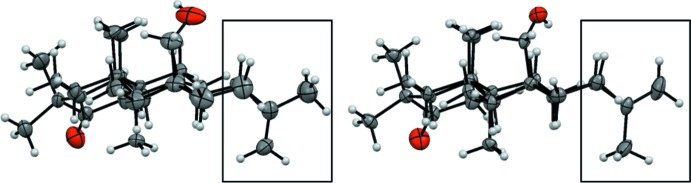
Different orientation of isopropenyl group virewd towards the C19/C20/C29/C30 plane in IA and IB.

## supplementary crystallographic information

### Comment

Betulone (lup-20 (29)-en-28-ol-3-one) also known as betulonic alcohol, is a pentacyclic triterpene of the lupane type which was first isolated as a natural product from *Betula lenta* in 1991 (Cole *et al.*, 1991). Betulone can be also isolated from various plants for example *Maytenus cuzcoina* and *Maytenus chiapensis* (Reyes *et al.*, 2006), *Betula alleghaniens* (Diouf *et al.*, 2009), *Excoecaria agallocha* (Liu *et al.*, 2010), *Ilex macropoda* (Kim *et al.*, 2002) and *Terminalia glabrescens* (Garcez *et al.*, 2003). The continually growing interest in betulone and its derivatives results from their wide spectrum of biological activities such as anti-inflammatory (Reyes *et al.*, 2006), anti-leishmanial (Alakurtti *et al.*, 2010) and anticancer (Hata *et al.*, 2002).

The structure of betulone is based on the 30-carbon skeleton comprising of four 6-membered rings and one cyclopentane ring. It has three available sites for simple chemical modification, namely: keto group at C3, primary hydroxy group at C28 and isopropenyl side chain at C19. These groups and their positions, mutual distances and orientation with respect to the rings can influence hydrogen bonding and the interactions of betulone with other active sites of surrounding species.

Betulone is also known as a derivative of betulin, which is one of the most plentiful triterpenes comprising up to 30% dry weight of the outer bark of the white birch. In comparison to betulin, the content of betulone in the outer bark of different tree species is very low, *e.g.* about of 0.03% in *Betula platyphylla* (Fuchino *et al.*, 1996) and for this reason the isolation from raw plant material is poorly profitable. A more effective method to obtain betulone with high yield is to carry out synthesis from betulin (Hase *et al.*, 1981). The crystal structure of betulone has not been reported until now. However, the crystal structures of betulonic acid-DMSO and betulonic acid-DMF solvates (Boryczka *et al.*, 2012*b*) were earlier described. In addition, the structure of betulinic acid-DMSO solvate (Boryczka *et al.*, 2012*a*) has also been reported. In the present work, we describe the crystal structure of betulone in order to gain a better understanding of the structure-activity relationships of this important molecule. Betulonic alcohol was obtained by oxidation of naturally occurring betulin in a one-step reaction utilizing *Jones*-oxidation (CrO_3_/H_2_SO_4_ in acetone-water solution) as the side product.

The asymmetric unit contains two independent molecules (IA and IB). A schematic drawing of the ring and atom labeling is shown in Fig. 1 and the asymmetric unit is shown in Fig. 2. All bond lengths and the angles show normal values. The cyclopentane ring adopts an envelope conformation and the cyclohexane rings are all in chair conformations. All the ring junctions in the lupane nucleus are *trans*-fused. A similar ring conformation is also observed in 20 (29)-lupene-3β,28β-diacetate (Mohamed *et al.*, 2006), 3,28-diacetoxy-29-bromo-betulin (Ding *et al.*, 2009) and also in betulin-ethanol (Drebushchak *et al.*, 2010), betulin-DMSO (Boryczka *et al.*, 2011), betulinic acid-DMSO (Boryczka *et al.*, 2012*a*) solvates. However, the conformation of ring A in betulone differs significantly from that observed earlier for betulonic acid-DMSO and DMF solvate, where ring A adopts a boat conformation. The molecules are packed along the *a* axis, in a zigzag fashion, parallel to the *bc* plane (Fig. 3). The cyclopentane ring is in an envelope conformation with the C17 atom being displaced from C18/C19/C21/C22 plane by 0.656 (4) Å (IA) and 0.674 (4) Å (IB). The C17—C18—C19—C21 and C19—C21—C22—C17 torsion angles are 27.0 (4)°, -25.4 (4)° (for IA) and 27.5 (3)°, -26.1 (3)° (for IB) respectively. The methyl groups C24, C25, C26, C27 occupy axial positions, but the methyl group C23 and isopropenyl group at C19 are equatorial. Fig. 4 shows the different orientations of the isopropenyl groups in the two independent molecules, (IA and IB). The value of the C21—C19—C20—C29 torsion angle describes the orientation of the isopropenyl group is equal to 92.3 (4)° (IA) and -98.8 (4)° (IB). The value of the C21—C19—C20—C29 torsion angle for betulin-DMSO and betulin-ethanol solvates are -96.8 (5)° and 88.6 (5)°, respectively. The hydroxymethyl group is attached to atom C17 of ring D in an axial orientation. No classical hydrogen bonding involving the hydroxy groups is observed. In the crystal, weak intermolecular C—H···O interactions link molecules into chains along [010]. Weak intramolecular C—H···O hydrogen bonds are also observed.

### Experimental

Betulonic alcohol was obtained by oxidation of naturally occurring betulin in one-step reaction utilizing *Jones*-oxidation (CrO_3_/H_2_SO_4_ in acetone-water solution) as the side product. The crude material was subjected to column chromatography on silica gel and eluted with CH_2_Cl_2_/C_2_H_5_OH (40:1, *v*/*v*). Betulonic alcohol [m.p. 367-369K, lit. m.p. 367-369K, Hata *et al.*, 2002), *R*_f_=0.68 (silica gel, CH_2_Cl_2_/C_2_H_5_OH, 40:1, *v*/*v*)] was crystallized from methanol, yielding colorless single crystals suitable for the X-ray analysis. ^1^H NMR (500 MHz, CDCl_3_) and EI MS data were identical with reported data (Hata *et al.*, 2002).

### Refinement

The aromatic hydrogen atoms were treated as riding on their parent carbon atoms with d(C—H) = 0.95 Å and assigned isotropic atomic displacement parameters equal to 1.2 times the value of the equivalent atomic displacement parameters of the parent carbon atom [*U*_iso_(H)= 1.2U_eq_(C)]. The methylene H atoms were constrained to an ideal geometry with d(C—H) = 0.99 A° or d(C—H) = 0.95 Å (for terminal methylene group) and *U*_iso_(H) = 1.2U_eq_(C). Methyl H atoms were constrained as riding atoms, fixed to the parent atoms with distance of 0.98 A° and *U*_iso_(H) = 1.5U_eq_(C). hydroxy H atoms were constrained as riding atoms with d(O—H) = 0.84 Å and *U*_iso_(H) = 1.5U_eq_(O). Hydrogen atoms involved in weak hydrogen bonds were refined freely with *U*_iso_(H) equal to 1.2U_eq_ of the parent atom. In the absence of significant anomalous dispersion effects the Friedel pairs were merged.

### Crystal data

**Table:**

C_30_H_48_O_2_	*F*(000) = 1952
*M**_r_* = 440.71	*D*_x_ = 1.149 Mg m^−^^3^
Orthorhombic, *P*2_1_2_1_2_1_	Mo *K*α radiation, λ = 0.71073 Å
Hall symbol: P 2ac 2ab	Cell parameters from 24992 reflections
*a* = 9.4447 (3) Å	θ = 2.5–34.6°
*b* = 19.1818 (6) Å	µ = 0.07 mm^−^^1^
*c* = 28.1141 (7) Å	*T* = 100 K
*V* = 5093.3 (3) Å^3^	Plate, colourless
*Z* = 8	0.60 × 0.56 × 0.20 mm

### Data collection

**Table:**

Oxford Diffraction diffractometer with a Sapphire3 detector	5036 independent reflections
Radiation source: fine-focus sealed tube	4401 reflections with *I* > 2σ(*I*)
Graphite monochromator	*R*_int_ = 0.055
Detector resolution: 16.0328 pixels mm^-1^	θ_max_ = 25.1°, θ_min_ = 2.5°
ω scan	*h* = −8→11
Absorption correction: multi-scan (*CrysAlis RED*; Oxford Diffraction, 2008)	*k* = −22→22
*T*_min_ = 0.960, *T*_max_ = 0.986	*l* = −33→33
61473 measured reflections	

### Refinement

**Table:**

Refinement on *F*^2^	Primary atom site location: structure-invariant direct methods
Least-squares matrix: full	Secondary atom site location: difference Fourier map
*R*[*F*^2^ > 2σ(*F*^2^)] = 0.051	Hydrogen site location: inferred from neighbouring sites
*wR*(*F*^2^) = 0.141	H atoms treated by a mixture of independent and constrained refinement
*S* = 1.03	*w* = 1/[σ^2^(*F*_o_^2^) + (0.1099*P*)^2^] where *P* = (*F*_o_^2^ + 2*F*_c_^2^)/3
5036 reflections	(Δ/σ)_max_ < 0.001
609 parameters	Δρ_max_ = 0.55 e Å^−^^3^
0 restraints	Δρ_min_ = −0.50 e Å^−^^3^

### Special details

**Table:**

Geometry. All e.s.d.'s (except the e.s.d. in the dihedral angle between two l.s. planes) are estimated using the full covariance matrix. The cell e.s.d.'s are taken into account individually in the estimation of e.s.d.'s in distances, angles and torsion angles; correlations between e.s.d.'s in cell parameters are only used when they are defined by crystal symmetry. An approximate (isotropic) treatment of cell e.s.d.'s is used for estimating e.s.d.'s involving l.s. planes.
Refinement. Refinement of *F*^2^ against ALL reflections. The weighted *R*-factor *wR* and goodness of fit *S* are based on *F*^2^, conventional *R*-factors *R* are based on *F*, with *F* set to zero for negative *F*^2^. The threshold expression of *F*^2^ > σ(*F*^2^) is used only for calculating *R*-factors(gt) *etc*. and is not relevant to the choice of reflections for refinement. *R*-factors based on *F*^2^ are statistically about twice as large as those based on *F*, and *R*- factors based on ALL data will be even larger.

### Fractional atomic coordinates and isotropic or equivalent isotropic displacement parameters (Å^2^)

**Table:**

	*x*	*y*	*z*	*U*_iso_*/*U*_eq_	
O1A	1.1431 (3)	0.77637 (14)	−0.03486 (10)	0.0509 (8)	
O2A	0.6372 (4)	0.28035 (18)	0.08472 (12)	0.0727 (11)	
H2A	0.7190	0.2945	0.0914	0.109*	
C1A	1.0227 (4)	0.60249 (18)	−0.04338 (11)	0.0299 (8)	
H1AA	0.9311	0.6232	−0.0531	0.036*	
H1AB	1.0404	0.5616	−0.0640	0.036*	
C2A	1.1415 (4)	0.6566 (2)	−0.05138 (13)	0.0385 (9)	
H2AA	1.2343	0.6339	−0.0461	0.046*	
H2AB	1.1383	0.6727	−0.0848	0.046*	
C3A	1.1299 (4)	0.71811 (19)	−0.01944 (13)	0.0320 (8)	
C4A	1.1037 (4)	0.70497 (18)	0.03356 (12)	0.0309 (8)	
C5A	0.9920 (3)	0.64490 (17)	0.03958 (11)	0.0255 (7)	
H5A	0.9010	0.6656	0.0282	0.031*	
C6A	0.9629 (4)	0.62503 (19)	0.09145 (11)	0.0329 (8)	
H6AA	1.0405	0.5951	0.1035	0.039*	
H6AB	0.9594	0.6677	0.1113	0.039*	
C7A	0.8220 (4)	0.58598 (19)	0.09519 (11)	0.0332 (8)	
H7AA	0.7447	0.6175	0.0850	0.040*	
H7AB	0.8050	0.5737	0.1289	0.040*	
C8A	0.8157 (4)	0.51877 (18)	0.06497 (10)	0.0267 (7)	
C9A	0.8698 (3)	0.53544 (17)	0.01340 (10)	0.0218 (7)	
H9A	0.7955	0.5666	−0.0005	0.026*	
C10A	1.0118 (3)	0.57800 (17)	0.00863 (11)	0.0251 (7)	
C11A	0.8664 (4)	0.46982 (17)	−0.01759 (11)	0.0264 (7)	
H11A	0.9318	0.4347	−0.0041	0.032*	
H11B	0.8998	0.4816	−0.0500	0.032*	
C12A	0.7174 (3)	0.43854 (17)	−0.02076 (10)	0.0249 (7)	
H12A	0.7214	0.3943	−0.0388	0.030*	
H12B	0.6548	0.4710	−0.0382	0.030*	
C13A	0.6552 (3)	0.42468 (17)	0.02894 (10)	0.0240 (7)	
H13A	0.723 (4)	0.3924 (18)	0.0478 (12)	0.029*	
C14A	0.6565 (3)	0.49143 (17)	0.06015 (10)	0.0244 (7)	
C15A	0.5923 (4)	0.4738 (2)	0.10962 (11)	0.0359 (9)	
H15A	0.6601	0.4439	0.1270	0.043*	
H15B	0.5823	0.5177	0.1278	0.043*	
C16A	0.4469 (4)	0.4367 (2)	0.10862 (12)	0.0374 (9)	
H16A	0.4186	0.4238	0.1414	0.045*	
H16B	0.3742	0.4686	0.0955	0.045*	
C17A	0.4564 (4)	0.37156 (19)	0.07795 (12)	0.0341 (8)	
C18A	0.5063 (4)	0.39119 (18)	0.02774 (11)	0.0268 (7)	
H18A	0.4399	0.4280	0.0160	0.032*	
C19A	0.4791 (4)	0.32596 (18)	−0.00271 (13)	0.0341 (8)	
H19A	0.558 (4)	0.296 (2)	0.0026 (13)	0.041*	
C20A	0.4436 (4)	0.33884 (19)	−0.05530 (13)	0.0352 (8)	
C21A	0.3519 (5)	0.2879 (2)	0.02319 (15)	0.0510 (11)	
H21A	0.2689	0.2846	0.0018	0.061*	
H21B	0.3799	0.2403	0.0331	0.061*	
C22A	0.3172 (5)	0.3332 (2)	0.06692 (15)	0.0489 (11)	
H22A	0.2406	0.3667	0.0596	0.059*	
H22B	0.2878	0.3039	0.0941	0.059*	
C23A	1.0466 (4)	0.77220 (19)	0.05630 (14)	0.0409 (9)	
H23A	1.1071	0.8115	0.0473	0.061*	
H23B	1.0465	0.7673	0.0910	0.061*	
H23C	0.9498	0.7807	0.0451	0.061*	
C24A	1.2508 (4)	0.6891 (2)	0.05663 (15)	0.0453 (10)	
H24A	1.2978	0.6518	0.0388	0.068*	
H24B	1.2372	0.6745	0.0897	0.068*	
H24C	1.3095	0.7312	0.0558	0.068*	
C25A	1.1442 (4)	0.5352 (2)	0.02094 (14)	0.0364 (8)	
H25A	1.1341	0.4879	0.0083	0.055*	
H25B	1.1554	0.5331	0.0556	0.055*	
H25C	1.2278	0.5574	0.0068	0.055*	
C26A	0.9099 (4)	0.46436 (19)	0.09009 (12)	0.0342 (8)	
H26A	1.0051	0.4836	0.0946	0.051*	
H26B	0.9155	0.4221	0.0705	0.051*	
H26C	0.8690	0.4528	0.1211	0.051*	
C27A	0.5578 (4)	0.54781 (17)	0.03828 (12)	0.0291 (8)	
H27A	0.4591	0.5325	0.0408	0.044*	
H27B	0.5823	0.5547	0.0047	0.044*	
H27C	0.5699	0.5918	0.0556	0.044*	
C28A	0.5492 (5)	0.3188 (2)	0.10332 (14)	0.0447 (10)	
H28A	0.6013	0.3454	0.1279	0.054*	
H28B	0.4835	0.2877	0.1207	0.054*	
C29A	0.3848 (4)	0.3965 (2)	−0.07222 (14)	0.0407 (9)	
H29A	0.3618	0.3998	−0.1050	0.049*	
H29B	0.3656	0.4345	−0.0515	0.049*	
C30A	0.4735 (6)	0.2800 (3)	−0.08691 (16)	0.0631 (13)	
H30A	0.4304	0.2886	−0.1181	0.095*	
H30B	0.4341	0.2373	−0.0732	0.095*	
H30C	0.5762	0.2749	−0.0906	0.095*	
O1B	−0.3496 (3)	0.80561 (15)	0.28884 (10)	0.0480 (7)	
O2B	0.6246 (3)	1.01860 (12)	0.13685 (8)	0.0344 (6)	
H2B	0.6040	1.0023	0.1637	0.052*	
C1B	0.0161 (4)	0.84111 (17)	0.28726 (11)	0.0261 (7)	
H1BA	0.1042	0.8236	0.3022	0.031*	
H1BB	−0.0123	0.8840	0.3044	0.031*	
C2B	−0.1003 (4)	0.78616 (18)	0.29336 (12)	0.0323 (8)	
H2BA	−0.0657	0.7410	0.2809	0.039*	
H2BB	−0.1203	0.7802	0.3277	0.039*	
C3B	−0.2362 (4)	0.80529 (17)	0.26795 (12)	0.0286 (7)	
C4B	−0.2235 (3)	0.82674 (16)	0.21577 (11)	0.0222 (7)	
C5B	−0.0983 (3)	0.88076 (15)	0.21148 (11)	0.0185 (6)	
H5B	−0.1308	0.9221	0.2302	0.022*	
C6B	−0.0769 (3)	0.90864 (16)	0.16122 (10)	0.0205 (6)	
H6BA	−0.1700	0.9196	0.1469	0.025*	
H6BB	−0.0307	0.8725	0.1414	0.025*	
C7B	0.0147 (3)	0.97415 (15)	0.16204 (10)	0.0197 (6)	
H7BA	−0.0361	1.0111	0.1798	0.024*	
H7BB	0.0278	0.9908	0.1290	0.024*	
C8B	0.1626 (3)	0.96323 (15)	0.18508 (10)	0.0164 (6)	
C9B	0.1422 (3)	0.92689 (14)	0.23454 (10)	0.0175 (6)	
H9BA	0.0894	0.9616	0.2543	0.021*	
C10B	0.0471 (3)	0.85976 (15)	0.23497 (10)	0.0191 (6)	
C11B	0.2853 (3)	0.91725 (16)	0.26026 (10)	0.0211 (6)	
H11C	0.2675	0.8986	0.2925	0.025*	
H11D	0.3423	0.8824	0.2427	0.025*	
C12B	0.3709 (3)	0.98473 (15)	0.26455 (10)	0.0214 (6)	
H12C	0.3226	1.0167	0.2869	0.026*	
H12D	0.4656	0.9739	0.2777	0.026*	
C13B	0.3881 (3)	1.02087 (15)	0.21660 (10)	0.0173 (6)	
H13B	0.435 (3)	0.9873 (17)	0.1934 (11)	0.021*	
C14B	0.2394 (3)	1.03694 (15)	0.19426 (10)	0.0177 (6)	
C15B	0.2574 (3)	1.07779 (16)	0.14691 (10)	0.0212 (6)	
H15C	0.2956	1.0456	0.1226	0.025*	
H15D	0.1627	1.0932	0.1360	0.025*	
C16B	0.3544 (3)	1.14170 (16)	0.14973 (11)	0.0242 (7)	
H16C	0.3116	1.1772	0.1708	0.029*	
H16D	0.3656	1.1624	0.1177	0.029*	
C17B	0.4999 (3)	1.11996 (15)	0.16926 (10)	0.0212 (7)	
C18B	0.4813 (3)	1.08681 (15)	0.21843 (10)	0.0200 (6)	
H18B	0.4304	1.1216	0.2387	0.024*	
C19B	0.6342 (3)	1.08099 (16)	0.23816 (11)	0.0218 (6)	
H19B	0.677 (4)	1.0405 (19)	0.2266 (12)	0.026*	
C20B	0.6521 (4)	1.08491 (17)	0.29161 (12)	0.0287 (7)	
C21B	0.7161 (4)	1.14185 (17)	0.21277 (12)	0.0276 (7)	
H21C	0.7537	1.1751	0.2365	0.033*	
H21D	0.7962	1.1232	0.1940	0.033*	
C22B	0.6084 (4)	1.17815 (17)	0.18003 (12)	0.0262 (7)	
H22C	0.551 (4)	1.2189 (18)	0.1957 (12)	0.031*	
H22D	0.662 (4)	1.1975 (18)	0.1499 (13)	0.031*	
C23B	−0.3632 (3)	0.86105 (17)	0.20088 (13)	0.0296 (7)	
H23D	−0.4416	0.8286	0.2066	0.044*	
H23E	−0.3594	0.8729	0.1670	0.044*	
H23F	−0.3779	0.9036	0.2195	0.044*	
C24B	−0.2054 (4)	0.76017 (17)	0.18541 (13)	0.0297 (8)	
H24D	−0.1275	0.7322	0.1982	0.045*	
H24E	−0.1842	0.7732	0.1525	0.045*	
H24F	−0.2931	0.7329	0.1863	0.045*	
C25B	0.1199 (3)	0.79668 (15)	0.21113 (12)	0.0237 (7)	
H25D	0.2216	0.7979	0.2178	0.035*	
H25E	0.1047	0.7987	0.1767	0.035*	
H25F	0.0794	0.7534	0.2237	0.035*	
C26B	0.2521 (3)	0.91803 (16)	0.15102 (10)	0.0218 (6)	
H26D	0.1926	0.8813	0.1374	0.033*	
H26E	0.3306	0.8968	0.1687	0.033*	
H26F	0.2899	0.9473	0.1254	0.033*	
C27B	0.1513 (3)	1.08464 (15)	0.22700 (11)	0.0237 (7)	
H27D	0.2041	1.1276	0.2334	0.036*	
H27E	0.1322	1.0605	0.2570	0.036*	
H27F	0.0615	1.0961	0.2114	0.036*	
C28B	0.5697 (3)	1.07459 (16)	0.13205 (11)	0.0231 (7)	
H28C	0.4967	1.0677	0.1072	0.028*	
H28D	0.6433	1.1044	0.1174	0.028*	
C29B	0.7480 (5)	1.0447 (2)	0.31341 (14)	0.0483 (11)	
H29C	0.7636	1.0495	0.3466	0.058*	
H29D	0.8004	1.0114	0.2957	0.058*	
C30B	0.5679 (5)	1.1375 (2)	0.31777 (13)	0.0491 (11)	
H30D	0.6030	1.1412	0.3505	0.074*	
H30E	0.4682	1.1233	0.3182	0.074*	
H30F	0.5768	1.1828	0.3020	0.074*	

### Atomic displacement parameters (Å^2^)

**Table:**

	*U*^11^	*U*^22^	*U*^33^	*U*^12^	*U*^13^	*U*^23^
O1A	0.0601 (19)	0.0390 (16)	0.0537 (18)	−0.0081 (14)	0.0087 (15)	−0.0007 (13)
O2A	0.082 (2)	0.081 (2)	0.055 (2)	0.034 (2)	0.027 (2)	0.0391 (18)
C1A	0.0320 (18)	0.0340 (18)	0.0237 (16)	−0.0067 (16)	0.0070 (14)	−0.0063 (14)
C2A	0.042 (2)	0.044 (2)	0.0295 (18)	−0.0090 (19)	0.0083 (17)	−0.0069 (16)
C3A	0.0208 (17)	0.034 (2)	0.041 (2)	−0.0040 (15)	−0.0020 (15)	−0.0043 (16)
C4A	0.0280 (17)	0.0333 (19)	0.0314 (18)	0.0025 (15)	−0.0057 (15)	−0.0109 (15)
C5A	0.0208 (16)	0.0335 (17)	0.0223 (16)	0.0038 (14)	−0.0046 (13)	−0.0046 (13)
C6A	0.0369 (19)	0.042 (2)	0.0191 (16)	0.0074 (16)	−0.0075 (14)	−0.0092 (15)
C7A	0.0346 (18)	0.049 (2)	0.0157 (15)	0.0052 (17)	0.0010 (14)	−0.0076 (15)
C8A	0.0298 (17)	0.0379 (19)	0.0125 (14)	0.0085 (15)	0.0004 (13)	0.0003 (13)
C9A	0.0215 (15)	0.0292 (17)	0.0145 (14)	0.0062 (14)	−0.0005 (12)	−0.0001 (12)
C10A	0.0228 (16)	0.0330 (18)	0.0195 (15)	0.0030 (14)	−0.0002 (13)	−0.0038 (13)
C11A	0.0289 (17)	0.0330 (18)	0.0173 (14)	0.0018 (15)	0.0046 (13)	−0.0011 (13)
C12A	0.0299 (17)	0.0297 (17)	0.0152 (15)	0.0001 (14)	0.0053 (13)	0.0011 (13)
C13A	0.0254 (16)	0.0304 (17)	0.0163 (14)	0.0050 (15)	0.0035 (13)	0.0064 (13)
C14A	0.0249 (16)	0.0348 (18)	0.0135 (14)	0.0058 (15)	0.0039 (12)	0.0038 (13)
C15A	0.040 (2)	0.053 (2)	0.0140 (15)	0.0062 (18)	0.0062 (14)	0.0019 (15)
C16A	0.0355 (19)	0.053 (2)	0.0235 (17)	0.0058 (18)	0.0145 (15)	0.0084 (16)
C17A	0.0359 (19)	0.041 (2)	0.0254 (17)	0.0050 (17)	0.0120 (15)	0.0129 (15)
C18A	0.0303 (18)	0.0296 (17)	0.0205 (16)	0.0043 (14)	0.0067 (14)	0.0097 (13)
C19A	0.037 (2)	0.0275 (18)	0.038 (2)	−0.0004 (16)	0.0083 (16)	0.0074 (15)
C20A	0.0259 (17)	0.041 (2)	0.038 (2)	−0.0069 (16)	0.0049 (15)	−0.0043 (16)
C21A	0.059 (3)	0.048 (2)	0.046 (2)	−0.016 (2)	0.015 (2)	0.0079 (19)
C22A	0.046 (2)	0.052 (2)	0.049 (2)	−0.007 (2)	0.022 (2)	0.016 (2)
C23A	0.040 (2)	0.037 (2)	0.046 (2)	0.0024 (18)	−0.0033 (18)	−0.0132 (17)
C24A	0.028 (2)	0.051 (2)	0.057 (3)	0.0003 (18)	−0.0116 (18)	−0.011 (2)
C25A	0.0232 (17)	0.038 (2)	0.048 (2)	0.0055 (16)	0.0005 (16)	−0.0091 (17)
C26A	0.0326 (19)	0.045 (2)	0.0247 (17)	0.0053 (17)	−0.0061 (14)	0.0085 (15)
C27A	0.0260 (17)	0.0286 (17)	0.0327 (18)	0.0050 (14)	−0.0003 (15)	0.0025 (14)
C28A	0.051 (2)	0.048 (2)	0.035 (2)	0.005 (2)	0.0139 (19)	0.0185 (18)
C29A	0.041 (2)	0.044 (2)	0.037 (2)	−0.0020 (18)	−0.0088 (17)	0.0047 (17)
C30A	0.074 (3)	0.071 (3)	0.044 (3)	0.019 (3)	0.008 (2)	−0.008 (2)
O1B	0.0398 (15)	0.0614 (18)	0.0428 (16)	−0.0095 (14)	0.0111 (13)	0.0146 (13)
O2B	0.0411 (14)	0.0317 (13)	0.0302 (12)	−0.0012 (12)	0.0049 (11)	0.0028 (10)
C1B	0.0302 (17)	0.0276 (16)	0.0206 (16)	−0.0045 (14)	0.0005 (13)	0.0040 (13)
C2B	0.040 (2)	0.0317 (18)	0.0254 (17)	−0.0080 (16)	−0.0008 (15)	0.0100 (14)
C3B	0.0295 (18)	0.0253 (17)	0.0310 (18)	−0.0106 (15)	0.0048 (15)	0.0057 (14)
C4B	0.0228 (16)	0.0197 (15)	0.0242 (16)	−0.0009 (13)	0.0003 (13)	0.0021 (13)
C5B	0.0184 (15)	0.0148 (14)	0.0222 (15)	−0.0008 (12)	0.0001 (12)	0.0008 (12)
C6B	0.0181 (15)	0.0229 (15)	0.0206 (15)	0.0020 (13)	−0.0040 (12)	0.0019 (12)
C7B	0.0232 (16)	0.0181 (14)	0.0177 (14)	0.0000 (13)	−0.0025 (12)	0.0055 (12)
C8B	0.0175 (14)	0.0178 (15)	0.0138 (13)	−0.0010 (12)	−0.0003 (11)	0.0023 (11)
C9B	0.0228 (15)	0.0154 (14)	0.0143 (13)	−0.0011 (13)	0.0006 (12)	0.0022 (11)
C10B	0.0226 (15)	0.0164 (14)	0.0185 (15)	−0.0007 (13)	−0.0016 (12)	0.0051 (11)
C11B	0.0272 (16)	0.0199 (15)	0.0162 (14)	−0.0015 (13)	−0.0017 (12)	0.0053 (12)
C12B	0.0257 (16)	0.0220 (15)	0.0165 (14)	−0.0007 (14)	−0.0022 (13)	0.0019 (12)
C13B	0.0201 (15)	0.0178 (14)	0.0139 (13)	−0.0010 (13)	−0.0006 (12)	−0.0019 (12)
C14B	0.0193 (15)	0.0156 (14)	0.0180 (14)	−0.0005 (13)	0.0001 (12)	0.0028 (12)
C15B	0.0224 (15)	0.0207 (15)	0.0206 (15)	−0.0008 (13)	−0.0015 (12)	0.0083 (13)
C16B	0.0285 (17)	0.0216 (15)	0.0225 (15)	−0.0010 (14)	−0.0019 (14)	0.0072 (12)
C17B	0.0248 (17)	0.0176 (15)	0.0211 (15)	−0.0007 (13)	−0.0009 (13)	0.0029 (12)
C18B	0.0257 (16)	0.0164 (14)	0.0180 (14)	−0.0009 (13)	−0.0019 (12)	0.0009 (12)
C19B	0.0228 (16)	0.0175 (15)	0.0250 (15)	−0.0039 (13)	−0.0040 (13)	−0.0005 (13)
C20B	0.0319 (18)	0.0265 (16)	0.0277 (17)	−0.0101 (15)	−0.0076 (14)	0.0008 (14)
C21B	0.0304 (17)	0.0230 (16)	0.0295 (17)	−0.0081 (14)	−0.0042 (14)	0.0010 (14)
C22B	0.0292 (17)	0.0193 (16)	0.0300 (17)	−0.0044 (14)	0.0006 (14)	0.0037 (13)
C23B	0.0208 (16)	0.0268 (17)	0.041 (2)	−0.0004 (14)	0.0019 (15)	0.0018 (14)
C24B	0.0271 (17)	0.0227 (17)	0.039 (2)	−0.0002 (14)	0.0004 (15)	−0.0040 (14)
C25B	0.0241 (16)	0.0159 (14)	0.0309 (17)	0.0001 (13)	−0.0053 (14)	0.0021 (13)
C26B	0.0270 (16)	0.0217 (15)	0.0166 (14)	−0.0026 (14)	0.0027 (13)	−0.0002 (12)
C27B	0.0268 (16)	0.0161 (14)	0.0282 (16)	0.0013 (13)	−0.0004 (13)	0.0017 (13)
C28B	0.0204 (15)	0.0269 (17)	0.0219 (16)	−0.0097 (15)	−0.0022 (13)	0.0015 (13)
C29B	0.065 (3)	0.047 (2)	0.033 (2)	−0.011 (2)	−0.022 (2)	0.0082 (17)
C30B	0.053 (3)	0.068 (3)	0.0266 (19)	−0.005 (2)	−0.0048 (18)	−0.0162 (19)

### Geometric parameters (Å, º)

**Table:**

O1A—C3A	1.205 (4)	O1B—C3B	1.221 (4)
O2A—C28A	1.228 (5)	O2B—C28B	1.200 (4)
O2A—H2A	0.8400	O2B—H2B	0.8400
C1A—C10A	1.539 (4)	C1B—C2B	1.533 (5)
C1A—C2A	1.546 (5)	C1B—C10B	1.541 (4)
C1A—H1AA	0.9900	C1B—H1BA	0.9900
C1A—H1AB	0.9900	C1B—H1BB	0.9900
C2A—C3A	1.486 (5)	C2B—C3B	1.514 (5)
C2A—H2AA	0.9900	C2B—H2BA	0.9900
C2A—H2AB	0.9900	C2B—H2BB	0.9900
C3A—C4A	1.531 (5)	C3B—C4B	1.528 (4)
C4A—C23A	1.537 (5)	C4B—C23B	1.533 (5)
C4A—C24A	1.562 (5)	C4B—C24B	1.546 (4)
C4A—C5A	1.572 (5)	C4B—C5B	1.577 (4)
C5A—C6A	1.532 (5)	C5B—C6B	1.524 (4)
C5A—C10A	1.562 (4)	C5B—C10B	1.576 (4)
C5A—H5A	1.0000	C5B—H5B	1.0000
C6A—C7A	1.531 (5)	C6B—C7B	1.526 (4)
C6A—H6AA	0.9900	C6B—H6BA	0.9900
C6A—H6AB	0.9900	C6B—H6BB	0.9900
C7A—C8A	1.545 (5)	C7B—C8B	1.554 (4)
C7A—H7AA	0.9900	C7B—H7BA	0.9900
C7A—H7AB	0.9900	C7B—H7BB	0.9900
C8A—C26A	1.543 (5)	C8B—C26B	1.543 (4)
C8A—C9A	1.570 (4)	C8B—C9B	1.567 (4)
C8A—C14A	1.598 (5)	C8B—C14B	1.610 (4)
C9A—C11A	1.531 (4)	C9B—C11B	1.544 (4)
C9A—C10A	1.576 (4)	C9B—C10B	1.570 (4)
C9A—H9A	1.0000	C9B—H9BA	1.0000
C10A—C25A	1.536 (5)	C10B—C25B	1.545 (4)
C11A—C12A	1.532 (5)	C11B—C12B	1.531 (4)
C11A—H11A	0.9900	C11B—H11C	0.9900
C11A—H11B	0.9900	C11B—H11D	0.9900
C12A—C13A	1.539 (4)	C12B—C13B	1.525 (4)
C12A—H12A	0.9900	C12B—H12C	0.9900
C12A—H12B	0.9900	C12B—H12D	0.9900
C13A—C18A	1.546 (5)	C13B—C18B	1.542 (4)
C13A—C14A	1.552 (4)	C13B—C14B	1.569 (4)
C13A—H13A	1.04 (4)	C13B—H13B	1.02 (3)
C14A—C27A	1.554 (4)	C14B—C27B	1.542 (4)
C14A—C15A	1.554 (4)	C14B—C15B	1.554 (4)
C15A—C16A	1.547 (5)	C15B—C16B	1.532 (4)
C15A—H15A	0.9900	C15B—H15C	0.9900
C15A—H15B	0.9900	C15B—H15D	0.9900
C16A—C17A	1.521 (5)	C16B—C17B	1.538 (4)
C16A—H16A	0.9900	C16B—H16C	0.9900
C16A—H16B	0.9900	C16B—H16D	0.9900
C17A—C28A	1.517 (5)	C17B—C28B	1.512 (4)
C17A—C18A	1.535 (4)	C17B—C18B	1.532 (4)
C17A—C22A	1.538 (6)	C17B—C22B	1.546 (4)
C18A—C19A	1.538 (5)	C18B—C19B	1.551 (4)
C18A—H18A	1.0000	C18B—H18B	1.0000
C19A—C20A	1.536 (5)	C19B—C20B	1.514 (4)
C19A—C21A	1.582 (5)	C19B—C21B	1.572 (4)
C19A—H19A	0.95 (4)	C19B—H19B	0.93 (4)
C20A—C29A	1.326 (5)	C20B—C29B	1.338 (5)
C20A—C30A	1.463 (6)	C20B—C30B	1.480 (5)
C21A—C22A	1.541 (6)	C21B—C22B	1.538 (5)
C21A—H21A	0.9900	C21B—H21C	0.9900
C21A—H21B	0.9900	C21B—H21D	0.9900
C22A—H22A	0.9900	C22B—H22C	1.05 (4)
C22A—H22B	0.9900	C22B—H22D	1.05 (4)
C23A—H23A	0.9800	C23B—H23D	0.9800
C23A—H23B	0.9800	C23B—H23E	0.9800
C23A—H23C	0.9800	C23B—H23F	0.9800
C24A—H24A	0.9800	C24B—H24D	0.9800
C24A—H24B	0.9800	C24B—H24E	0.9800
C24A—H24C	0.9800	C24B—H24F	0.9800
C25A—H25A	0.9800	C25B—H25D	0.9800
C25A—H25B	0.9800	C25B—H25E	0.9800
C25A—H25C	0.9800	C25B—H25F	0.9800
C26A—H26A	0.9800	C26B—H26D	0.9800
C26A—H26B	0.9800	C26B—H26E	0.9800
C26A—H26C	0.9800	C26B—H26F	0.9800
C27A—H27A	0.9800	C27B—H27D	0.9800
C27A—H27B	0.9800	C27B—H27E	0.9800
C27A—H27C	0.9800	C27B—H27F	0.9800
C28A—H28A	0.9900	C28B—H28C	0.9900
C28A—H28B	0.9900	C28B—H28D	0.9900
C29A—H29A	0.9500	C29B—H29C	0.9500
C29A—H29B	0.9500	C29B—H29D	0.9500
C30A—H30A	0.9800	C30B—H30D	0.9800
C30A—H30B	0.9800	C30B—H30E	0.9800
C30A—H30C	0.9800	C30B—H30F	0.9800
			
C28A—O2A—H2A	109.5	C28B—O2B—H2B	109.5
C10A—C1A—C2A	113.1 (3)	C2B—C1B—C10B	113.7 (3)
C10A—C1A—H1AA	109.0	C2B—C1B—H1BA	108.8
C2A—C1A—H1AA	109.0	C10B—C1B—H1BA	108.8
C10A—C1A—H1AB	109.0	C2B—C1B—H1BB	108.8
C2A—C1A—H1AB	109.0	C10B—C1B—H1BB	108.8
H1AA—C1A—H1AB	107.8	H1BA—C1B—H1BB	107.7
C3A—C2A—C1A	113.1 (3)	C3B—C2B—C1B	112.9 (3)
C3A—C2A—H2AA	109.0	C3B—C2B—H2BA	109.0
C1A—C2A—H2AA	109.0	C1B—C2B—H2BA	109.0
C3A—C2A—H2AB	109.0	C3B—C2B—H2BB	109.0
C1A—C2A—H2AB	109.0	C1B—C2B—H2BB	109.0
H2AA—C2A—H2AB	107.8	H2BA—C2B—H2BB	107.8
O1A—C3A—C2A	120.7 (3)	O1B—C3B—C2B	121.2 (3)
O1A—C3A—C4A	121.3 (3)	O1B—C3B—C4B	121.9 (3)
C2A—C3A—C4A	118.0 (3)	C2B—C3B—C4B	116.8 (3)
C3A—C4A—C23A	108.9 (3)	C3B—C4B—C23B	108.1 (3)
C3A—C4A—C24A	107.0 (3)	C3B—C4B—C24B	108.4 (3)
C23A—C4A—C24A	107.6 (3)	C23B—C4B—C24B	107.4 (3)
C3A—C4A—C5A	109.5 (3)	C3B—C4B—C5B	108.0 (2)
C23A—C4A—C5A	109.5 (3)	C23B—C4B—C5B	110.0 (2)
C24A—C4A—C5A	114.2 (3)	C24B—C4B—C5B	114.7 (3)
C6A—C5A—C10A	110.3 (3)	C6B—C5B—C10B	111.3 (2)
C6A—C5A—C4A	113.9 (3)	C6B—C5B—C4B	113.6 (2)
C10A—C5A—C4A	117.5 (3)	C10B—C5B—C4B	117.0 (2)
C6A—C5A—H5A	104.5	C6B—C5B—H5B	104.5
C10A—C5A—H5A	104.5	C10B—C5B—H5B	104.5
C4A—C5A—H5A	104.5	C4B—C5B—H5B	104.5
C7A—C6A—C5A	110.0 (3)	C5B—C6B—C7B	110.5 (2)
C7A—C6A—H6AA	109.7	C5B—C6B—H6BA	109.6
C5A—C6A—H6AA	109.7	C7B—C6B—H6BA	109.6
C7A—C6A—H6AB	109.7	C5B—C6B—H6BB	109.6
C5A—C6A—H6AB	109.7	C7B—C6B—H6BB	109.6
H6AA—C6A—H6AB	108.2	H6BA—C6B—H6BB	108.1
C6A—C7A—C8A	113.8 (3)	C6B—C7B—C8B	113.9 (2)
C6A—C7A—H7AA	108.8	C6B—C7B—H7BA	108.8
C8A—C7A—H7AA	108.8	C8B—C7B—H7BA	108.8
C6A—C7A—H7AB	108.8	C6B—C7B—H7BB	108.8
C8A—C7A—H7AB	108.8	C8B—C7B—H7BB	108.8
H7AA—C7A—H7AB	107.7	H7BA—C7B—H7BB	107.7
C26A—C8A—C7A	106.9 (3)	C26B—C8B—C7B	108.0 (2)
C26A—C8A—C9A	111.9 (3)	C26B—C8B—C9B	111.6 (2)
C7A—C8A—C9A	109.0 (3)	C7B—C8B—C9B	108.6 (2)
C26A—C8A—C14A	111.1 (3)	C26B—C8B—C14B	110.3 (2)
C7A—C8A—C14A	110.9 (3)	C7B—C8B—C14B	110.7 (2)
C9A—C8A—C14A	107.1 (2)	C9B—C8B—C14B	107.7 (2)
C11A—C9A—C8A	110.6 (3)	C11B—C9B—C8B	111.2 (2)
C11A—C9A—C10A	113.3 (2)	C11B—C9B—C10B	113.5 (2)
C8A—C9A—C10A	117.4 (2)	C8B—C9B—C10B	116.2 (2)
C11A—C9A—H9A	104.7	C11B—C9B—H9BA	104.9
C8A—C9A—H9A	104.7	C8B—C9B—H9BA	104.9
C10A—C9A—H9A	104.7	C10B—C9B—H9BA	104.9
C25A—C10A—C1A	108.8 (3)	C1B—C10B—C25B	108.5 (2)
C25A—C10A—C5A	114.3 (3)	C1B—C10B—C9B	107.8 (2)
C1A—C10A—C5A	106.6 (3)	C25B—C10B—C9B	112.6 (2)
C25A—C10A—C9A	113.4 (3)	C1B—C10B—C5B	107.1 (2)
C1A—C10A—C9A	107.2 (2)	C25B—C10B—C5B	113.9 (2)
C5A—C10A—C9A	106.0 (2)	C9B—C10B—C5B	106.6 (2)
C9A—C11A—C12A	112.0 (3)	C12B—C11B—C9B	113.5 (2)
C9A—C11A—H11A	109.2	C12B—C11B—H11C	108.9
C12A—C11A—H11A	109.2	C9B—C11B—H11C	108.9
C9A—C11A—H11B	109.2	C12B—C11B—H11D	108.9
C12A—C11A—H11B	109.2	C9B—C11B—H11D	108.9
H11A—C11A—H11B	107.9	H11C—C11B—H11D	107.7
C11A—C12A—C13A	111.4 (3)	C13B—C12B—C11B	111.8 (2)
C11A—C12A—H12A	109.3	C13B—C12B—H12C	109.3
C13A—C12A—H12A	109.3	C11B—C12B—H12C	109.3
C11A—C12A—H12B	109.3	C13B—C12B—H12D	109.3
C13A—C12A—H12B	109.3	C11B—C12B—H12D	109.3
H12A—C12A—H12B	108.0	H12C—C12B—H12D	107.9
C12A—C13A—C18A	113.5 (3)	C12B—C13B—C18B	113.9 (2)
C12A—C13A—C14A	111.6 (3)	C12B—C13B—C14B	110.3 (2)
C18A—C13A—C14A	111.2 (3)	C18B—C13B—C14B	111.3 (2)
C12A—C13A—H13A	109.4 (19)	C12B—C13B—H13B	109.1 (17)
C18A—C13A—H13A	108.9 (19)	C18B—C13B—H13B	106.8 (18)
C14A—C13A—H13A	101.5 (19)	C14B—C13B—H13B	105.0 (18)
C13A—C14A—C27A	110.2 (3)	C27B—C14B—C15B	105.7 (2)
C13A—C14A—C15A	108.9 (3)	C27B—C14B—C13B	111.2 (2)
C27A—C14A—C15A	105.7 (3)	C15B—C14B—C13B	110.1 (2)
C13A—C14A—C8A	109.0 (2)	C27B—C14B—C8B	112.0 (2)
C27A—C14A—C8A	111.7 (3)	C15B—C14B—C8B	110.8 (2)
C15A—C14A—C8A	111.2 (3)	C13B—C14B—C8B	107.1 (2)
C16A—C15A—C14A	115.5 (3)	C16B—C15B—C14B	115.1 (2)
C16A—C15A—H15A	108.4	C16B—C15B—H15C	108.5
C14A—C15A—H15A	108.4	C14B—C15B—H15C	108.5
C16A—C15A—H15B	108.4	C16B—C15B—H15D	108.5
C14A—C15A—H15B	108.4	C14B—C15B—H15D	108.5
H15A—C15A—H15B	107.5	H15C—C15B—H15D	107.5
C17A—C16A—C15A	109.6 (3)	C15B—C16B—C17B	109.6 (2)
C17A—C16A—H16A	109.7	C15B—C16B—H16C	109.7
C15A—C16A—H16A	109.7	C17B—C16B—H16C	109.7
C17A—C16A—H16B	109.7	C15B—C16B—H16D	109.7
C15A—C16A—H16B	109.7	C17B—C16B—H16D	109.7
H16A—C16A—H16B	108.2	H16C—C16B—H16D	108.2
C28A—C17A—C16A	108.4 (3)	C28B—C17B—C18B	115.8 (2)
C28A—C17A—C18A	114.7 (3)	C28B—C17B—C16B	107.4 (3)
C16A—C17A—C18A	109.7 (3)	C18B—C17B—C16B	109.4 (2)
C28A—C17A—C22A	105.6 (3)	C28B—C17B—C22B	105.2 (3)
C16A—C17A—C22A	117.2 (3)	C18B—C17B—C22B	101.5 (2)
C18A—C17A—C22A	101.2 (3)	C16B—C17B—C22B	117.8 (2)
C17A—C18A—C19A	105.1 (3)	C17B—C18B—C13B	112.1 (2)
C17A—C18A—C13A	111.2 (3)	C17B—C18B—C19B	104.2 (2)
C19A—C18A—C13A	120.2 (3)	C13B—C18B—C19B	119.0 (2)
C17A—C18A—H18A	106.5	C17B—C18B—H18B	107.0
C19A—C18A—H18A	106.5	C13B—C18B—H18B	107.0
C13A—C18A—H18A	106.5	C19B—C18B—H18B	107.0
C20A—C19A—C18A	116.2 (3)	C20B—C19B—C18B	117.1 (3)
C20A—C19A—C21A	110.6 (3)	C20B—C19B—C21B	111.0 (3)
C18A—C19A—C21A	104.2 (3)	C18B—C19B—C21B	104.0 (2)
C20A—C19A—H19A	115 (2)	C20B—C19B—H19B	110 (2)
C18A—C19A—H19A	105 (2)	C18B—C19B—H19B	110 (2)
C21A—C19A—H19A	104 (2)	C21B—C19B—H19B	104 (2)
C29A—C20A—C30A	120.4 (4)	C29B—C20B—C30B	121.9 (3)
C29A—C20A—C19A	124.8 (3)	C29B—C20B—C19B	120.1 (3)
C30A—C20A—C19A	114.7 (3)	C30B—C20B—C19B	117.8 (3)
C22A—C21A—C19A	105.6 (3)	C22B—C21B—C19B	106.4 (3)
C22A—C21A—H21A	110.6	C22B—C21B—H21C	110.4
C19A—C21A—H21A	110.6	C19B—C21B—H21C	110.4
C22A—C21A—H21B	110.6	C22B—C21B—H21D	110.4
C19A—C21A—H21B	110.6	C19B—C21B—H21D	110.4
H21A—C21A—H21B	108.8	H21C—C21B—H21D	108.6
C17A—C22A—C21A	104.4 (3)	C21B—C22B—C17B	103.2 (2)
C17A—C22A—H22A	110.9	C21B—C22B—H22C	115.4 (19)
C21A—C22A—H22A	110.9	C17B—C22B—H22C	106 (2)
C17A—C22A—H22B	110.9	C21B—C22B—H22D	109 (2)
C21A—C22A—H22B	110.9	C17B—C22B—H22D	114.5 (19)
H22A—C22A—H22B	108.9	H22C—C22B—H22D	109 (3)
C4A—C23A—H23A	109.5	C4B—C23B—H23D	109.5
C4A—C23A—H23B	109.5	C4B—C23B—H23E	109.5
H23A—C23A—H23B	109.5	H23D—C23B—H23E	109.5
C4A—C23A—H23C	109.5	C4B—C23B—H23F	109.5
H23A—C23A—H23C	109.5	H23D—C23B—H23F	109.5
H23B—C23A—H23C	109.5	H23E—C23B—H23F	109.5
C4A—C24A—H24A	109.5	C4B—C24B—H24D	109.5
C4A—C24A—H24B	109.5	C4B—C24B—H24E	109.5
H24A—C24A—H24B	109.5	H24D—C24B—H24E	109.5
C4A—C24A—H24C	109.5	C4B—C24B—H24F	109.5
H24A—C24A—H24C	109.5	H24D—C24B—H24F	109.5
H24B—C24A—H24C	109.5	H24E—C24B—H24F	109.5
C10A—C25A—H25A	109.5	C10B—C25B—H25D	109.5
C10A—C25A—H25B	109.5	C10B—C25B—H25E	109.5
H25A—C25A—H25B	109.5	H25D—C25B—H25E	109.5
C10A—C25A—H25C	109.5	C10B—C25B—H25F	109.5
H25A—C25A—H25C	109.5	H25D—C25B—H25F	109.5
H25B—C25A—H25C	109.5	H25E—C25B—H25F	109.5
C8A—C26A—H26A	109.5	C8B—C26B—H26D	109.5
C8A—C26A—H26B	109.5	C8B—C26B—H26E	109.5
H26A—C26A—H26B	109.5	H26D—C26B—H26E	109.5
C8A—C26A—H26C	109.5	C8B—C26B—H26F	109.5
H26A—C26A—H26C	109.5	H26D—C26B—H26F	109.5
H26B—C26A—H26C	109.5	H26E—C26B—H26F	109.5
C14A—C27A—H27A	109.5	C14B—C27B—H27D	109.5
C14A—C27A—H27B	109.5	C14B—C27B—H27E	109.5
H27A—C27A—H27B	109.5	H27D—C27B—H27E	109.5
C14A—C27A—H27C	109.5	C14B—C27B—H27F	109.5
H27A—C27A—H27C	109.5	H27D—C27B—H27F	109.5
H27B—C27A—H27C	109.5	H27E—C27B—H27F	109.5
O2A—C28A—C17A	126.3 (4)	O2B—C28B—C17B	128.7 (3)
O2A—C28A—H28A	105.8	O2B—C28B—H28C	105.1
C17A—C28A—H28A	105.8	C17B—C28B—H28C	105.1
O2A—C28A—H28B	105.8	O2B—C28B—H28D	105.1
C17A—C28A—H28B	105.8	C17B—C28B—H28D	105.1
H28A—C28A—H28B	106.2	H28C—C28B—H28D	105.9
C20A—C29A—H29A	120.0	C20B—C29B—H29C	120.0
C20A—C29A—H29B	120.0	C20B—C29B—H29D	120.0
H29A—C29A—H29B	120.0	H29C—C29B—H29D	120.0
C20A—C30A—H30A	109.5	C20B—C30B—H30D	109.5
C20A—C30A—H30B	109.5	C20B—C30B—H30E	109.5
H30A—C30A—H30B	109.5	H30D—C30B—H30E	109.5
C20A—C30A—H30C	109.5	C20B—C30B—H30F	109.5
H30A—C30A—H30C	109.5	H30D—C30B—H30F	109.5
H30B—C30A—H30C	109.5	H30E—C30B—H30F	109.5
			
C10A—C1A—C2A—C3A	−54.6 (4)	C10B—C1B—C2B—C3B	−53.5 (4)
C1A—C2A—C3A—O1A	−134.1 (4)	C1B—C2B—C3B—O1B	−127.3 (4)
C1A—C2A—C3A—C4A	47.1 (4)	C1B—C2B—C3B—C4B	50.4 (4)
O1A—C3A—C4A—C23A	20.0 (5)	O1B—C3B—C4B—C23B	11.8 (4)
C2A—C3A—C4A—C23A	−161.2 (3)	C2B—C3B—C4B—C23B	−165.9 (3)
O1A—C3A—C4A—C24A	−96.0 (4)	O1B—C3B—C4B—C24B	−104.3 (4)
C2A—C3A—C4A—C24A	82.8 (4)	C2B—C3B—C4B—C24B	78.0 (3)
O1A—C3A—C4A—C5A	139.8 (3)	O1B—C3B—C4B—C5B	130.8 (3)
C2A—C3A—C4A—C5A	−41.5 (4)	C2B—C3B—C4B—C5B	−46.9 (4)
C3A—C4A—C5A—C6A	177.3 (3)	C3B—C4B—C5B—C6B	−178.0 (3)
C23A—C4A—C5A—C6A	−63.4 (4)	C23B—C4B—C5B—C6B	−60.2 (3)
C24A—C4A—C5A—C6A	57.3 (4)	C24B—C4B—C5B—C6B	61.0 (3)
C3A—C4A—C5A—C10A	46.0 (4)	C3B—C4B—C5B—C10B	50.2 (3)
C23A—C4A—C5A—C10A	165.3 (3)	C23B—C4B—C5B—C10B	167.9 (3)
C24A—C4A—C5A—C10A	−73.9 (4)	C24B—C4B—C5B—C10B	−70.9 (3)
C10A—C5A—C6A—C7A	−64.5 (3)	C10B—C5B—C6B—C7B	−61.5 (3)
C4A—C5A—C6A—C7A	160.8 (3)	C4B—C5B—C6B—C7B	164.0 (2)
C5A—C6A—C7A—C8A	58.6 (4)	C5B—C6B—C7B—C8B	57.8 (3)
C6A—C7A—C8A—C26A	72.9 (4)	C6B—C7B—C8B—C26B	70.7 (3)
C6A—C7A—C8A—C9A	−48.2 (4)	C6B—C7B—C8B—C9B	−50.5 (3)
C6A—C7A—C8A—C14A	−165.8 (3)	C6B—C7B—C8B—C14B	−168.5 (2)
C26A—C8A—C9A—C11A	61.5 (4)	C26B—C8B—C9B—C11B	63.4 (3)
C7A—C8A—C9A—C11A	179.5 (3)	C7B—C8B—C9B—C11B	−177.7 (2)
C14A—C8A—C9A—C11A	−60.5 (3)	C14B—C8B—C9B—C11B	−57.8 (3)
C26A—C8A—C9A—C10A	−70.6 (4)	C26B—C8B—C9B—C10B	−68.5 (3)
C7A—C8A—C9A—C10A	47.4 (4)	C7B—C8B—C9B—C10B	50.4 (3)
C14A—C8A—C9A—C10A	167.4 (2)	C14B—C8B—C9B—C10B	170.3 (2)
C2A—C1A—C10A—C25A	−68.0 (4)	C2B—C1B—C10B—C25B	−69.9 (3)
C2A—C1A—C10A—C5A	55.7 (4)	C2B—C1B—C10B—C9B	167.9 (3)
C2A—C1A—C10A—C9A	169.0 (3)	C2B—C1B—C10B—C5B	53.5 (3)
C6A—C5A—C10A—C25A	−66.3 (4)	C11B—C9B—C10B—C1B	60.4 (3)
C4A—C5A—C10A—C25A	66.6 (4)	C8B—C9B—C10B—C1B	−168.8 (2)
C6A—C5A—C10A—C1A	173.5 (3)	C11B—C9B—C10B—C25B	−59.2 (3)
C4A—C5A—C10A—C1A	−53.7 (3)	C8B—C9B—C10B—C25B	71.6 (3)
C6A—C5A—C10A—C9A	59.4 (3)	C11B—C9B—C10B—C5B	175.1 (2)
C4A—C5A—C10A—C9A	−167.7 (3)	C8B—C9B—C10B—C5B	−54.1 (3)
C11A—C9A—C10A—C25A	−57.7 (3)	C6B—C5B—C10B—C1B	173.1 (2)
C8A—C9A—C10A—C25A	73.2 (3)	C4B—C5B—C10B—C1B	−54.0 (3)
C11A—C9A—C10A—C1A	62.5 (3)	C6B—C5B—C10B—C25B	−66.9 (3)
C8A—C9A—C10A—C1A	−166.6 (3)	C4B—C5B—C10B—C25B	66.0 (3)
C11A—C9A—C10A—C5A	176.1 (2)	C6B—C5B—C10B—C9B	57.9 (3)
C8A—C9A—C10A—C5A	−53.0 (3)	C4B—C5B—C10B—C9B	−169.2 (2)
C8A—C9A—C11A—C12A	58.3 (3)	C8B—C9B—C11B—C12B	53.0 (3)
C10A—C9A—C11A—C12A	−167.5 (3)	C10B—C9B—C11B—C12B	−173.7 (2)
C9A—C11A—C12A—C13A	−54.3 (4)	C9B—C11B—C12B—C13B	−52.1 (3)
C11A—C12A—C13A—C18A	−178.4 (3)	C11B—C12B—C13B—C18B	−176.5 (2)
C11A—C12A—C13A—C14A	54.9 (4)	C11B—C12B—C13B—C14B	57.5 (3)
C12A—C13A—C14A—C27A	64.4 (3)	C12B—C13B—C14B—C27B	59.7 (3)
C18A—C13A—C14A—C27A	−63.5 (3)	C18B—C13B—C14B—C27B	−67.7 (3)
C12A—C13A—C14A—C15A	179.9 (3)	C12B—C13B—C14B—C15B	176.5 (2)
C18A—C13A—C14A—C15A	52.0 (3)	C18B—C13B—C14B—C15B	49.1 (3)
C12A—C13A—C14A—C8A	−58.5 (3)	C12B—C13B—C14B—C8B	−62.9 (3)
C18A—C13A—C14A—C8A	173.6 (2)	C18B—C13B—C14B—C8B	169.7 (2)
C26A—C8A—C14A—C13A	−62.2 (3)	C26B—C8B—C14B—C27B	178.3 (2)
C7A—C8A—C14A—C13A	179.1 (2)	C7B—C8B—C14B—C27B	58.9 (3)
C9A—C8A—C14A—C13A	60.3 (3)	C9B—C8B—C14B—C27B	−59.7 (3)
C26A—C8A—C14A—C27A	175.8 (3)	C26B—C8B—C14B—C15B	60.6 (3)
C7A—C8A—C14A—C27A	57.1 (3)	C7B—C8B—C14B—C15B	−58.9 (3)
C9A—C8A—C14A—C27A	−61.7 (3)	C9B—C8B—C14B—C15B	−177.5 (2)
C26A—C8A—C14A—C15A	57.9 (3)	C26B—C8B—C14B—C13B	−59.6 (3)
C7A—C8A—C14A—C15A	−60.8 (3)	C7B—C8B—C14B—C13B	−179.0 (2)
C9A—C8A—C14A—C15A	−179.6 (3)	C9B—C8B—C14B—C13B	62.4 (3)
C13A—C14A—C15A—C16A	−51.1 (4)	C27B—C14B—C15B—C16B	70.0 (3)
C27A—C14A—C15A—C16A	67.3 (4)	C13B—C14B—C15B—C16B	−50.2 (3)
C8A—C14A—C15A—C16A	−171.3 (3)	C8B—C14B—C15B—C16B	−168.5 (2)
C14A—C15A—C16A—C17A	54.3 (4)	C14B—C15B—C16B—C17B	55.1 (3)
C15A—C16A—C17A—C28A	69.0 (3)	C15B—C16B—C17B—C28B	68.1 (3)
C15A—C16A—C17A—C18A	−57.0 (4)	C15B—C16B—C17B—C18B	−58.4 (3)
C15A—C16A—C17A—C22A	−171.7 (3)	C15B—C16B—C17B—C22B	−173.5 (3)
C28A—C17A—C18A—C19A	70.4 (4)	C28B—C17B—C18B—C13B	−60.6 (3)
C16A—C17A—C18A—C19A	−167.2 (3)	C16B—C17B—C18B—C13B	60.9 (3)
C22A—C17A—C18A—C19A	−42.8 (3)	C22B—C17B—C18B—C13B	−173.8 (2)
C28A—C17A—C18A—C13A	−61.1 (4)	C28B—C17B—C18B—C19B	69.4 (3)
C16A—C17A—C18A—C13A	61.3 (4)	C16B—C17B—C18B—C19B	−169.1 (2)
C22A—C17A—C18A—C13A	−174.3 (3)	C22B—C17B—C18B—C19B	−43.9 (3)
C12A—C13A—C18A—C17A	174.0 (3)	C12B—C13B—C18B—C17B	178.0 (3)
C14A—C13A—C18A—C17A	−59.1 (3)	C14B—C13B—C18B—C17B	−56.5 (3)
C12A—C13A—C18A—C19A	50.7 (4)	C12B—C13B—C18B—C19B	56.2 (4)
C14A—C13A—C18A—C19A	177.6 (3)	C14B—C13B—C18B—C19B	−178.4 (2)
C17A—C18A—C19A—C20A	149.0 (3)	C17B—C18B—C19B—C20B	150.4 (3)
C13A—C18A—C19A—C20A	−84.9 (4)	C13B—C18B—C19B—C20B	−83.9 (3)
C17A—C18A—C19A—C21A	27.0 (4)	C17B—C18B—C19B—C21B	27.5 (3)
C13A—C18A—C19A—C21A	153.2 (3)	C13B—C18B—C19B—C21B	153.2 (3)
C18A—C19A—C20A—C29A	−26.2 (5)	C18B—C19B—C20B—C29B	142.0 (3)
C21A—C19A—C20A—C29A	92.3 (4)	C21B—C19B—C20B—C29B	−98.8 (4)
C18A—C19A—C20A—C30A	156.6 (4)	C18B—C19B—C20B—C30B	−42.1 (4)
C21A—C19A—C20A—C30A	−84.8 (4)	C21B—C19B—C20B—C30B	77.1 (4)
C20A—C19A—C21A—C22A	−126.5 (4)	C20B—C19B—C21B—C22B	−127.4 (3)
C18A—C19A—C21A—C22A	−0.9 (4)	C18B—C19B—C21B—C22B	−0.6 (3)
C28A—C17A—C22A—C21A	−78.2 (4)	C19B—C21B—C22B—C17B	−26.1 (3)
C16A—C17A—C22A—C21A	161.0 (3)	C28B—C17B—C22B—C21B	−78.0 (3)
C18A—C17A—C22A—C21A	41.7 (4)	C18B—C17B—C22B—C21B	43.0 (3)
C19A—C21A—C22A—C17A	−25.4 (4)	C16B—C17B—C22B—C21B	162.4 (3)
C16A—C17A—C28A—O2A	−140.3 (5)	C16B—C17B—C28B—O2B	−130.4 (3)
C18A—C17A—C28A—O2A	−17.2 (6)	C18B—C17B—C28B—O2B	−7.8 (5)
C22A—C17A—C28A—O2A	93.3 (5)	C22B—C17B—C28B—O2B	103.3 (4)

### Hydrogen-bond geometry (Å, º)

**Table:**

*D*—H···*A*	*D*—H	H···*A*	*D*···*A*	*D*—H···*A*
C13*A*—H13*A*···O2*A*	1.04 (4)	2.52 (3)	3.186 (4)	122 (2)
C13*B*—H13*B*···O2*B*	1.02 (3)	2.47 (3)	3.165 (4)	125 (2)
C19*A*—H19*A*···O2*A*	0.95 (4)	2.45 (4)	3.006 (5)	118 (3)
C22*B*—H22*C*···O1*B*^i^	1.05 (4)	2.56 (4)	3.567 (4)	160 (3)

Symmetry code: (i) −*x*, *y*+1/2, −*z*+1/2.
